# Opportunities to Increase Access to HIV Prevention: Evaluating the Implementation of Pharmacist-Initiated Pre-exposure Prophylaxis in California

**DOI:** 10.1093/ofid/ofad549

**Published:** 2023-11-03

**Authors:** Lauren A Hunter, Laura J Packel, Pooja Chitle, Raiza M Beltran, Sally Rafie, Loriann De Martini, Betty Dong, Orlando Harris, Ian W Holloway, Ayako Miyashita Ochoa, Sandra I McCoy

**Affiliations:** School of Public Health, Division of Epidemiology, University of California, Berkeley, Berkeley, California, USA; School of Public Health, Division of Epidemiology, University of California, Berkeley, Berkeley, California, USA; School of Public Health, Division of Epidemiology, University of California, Berkeley, Berkeley, California, USA; Luskin School of Public Affairs, University of California, Los Angeles, Los Angeles, California, USA; Birth Control Pharmacist, San Diego, California, USA; University of California, San Diego Health, San Diego, California, USA; California Society of Health-System Pharmacists, Sacramento, California, USA; School of Pharmacy, University of California, San Francisco, San Francisco, California, USA; School of Nursing, University of California, San Francisco, San Francisco, California, USA; Luskin School of Public Affairs, University of California, Los Angeles, Los Angeles, California, USA; Luskin School of Public Affairs, University of California, Los Angeles, Los Angeles, California, USA; School of Public Health, Division of Epidemiology, University of California, Berkeley, Berkeley, California, USA

**Keywords:** California, HIV prevention, pharmacies, post-exposure prophylaxis, pre-exposure prophylaxis

## Abstract

**Background:**

Pharmacies are a promising setting through which to expand access to human immunodeficiency virus (HIV) prevention, including pre-exposure and post-exposure prophylaxis (PrEP and PEP, respectively). We aimed to evaluate and inform the implementation of California's Senate Bill 159 (2019), allowing pharmacists to independently prescribe PrEP and PEP.

**Methods:**

From October through December 2022, we conducted a cross-sectional study of 919 California pharmacists and pharmacy students, primarily recruited via the email listservs of professional organizations. Participants completed an online survey assessing the implementation of pharmacist-initiated PrEP/PEP, including knowledge, attitudes, practices, perceived barriers, and implementation preferences elicited through a discrete choice experiment.

**Results:**

Among 919 participants (84% practicing pharmacists, 43% in community pharmacies), 11% and 13% reported that pharmacists at their pharmacy initiate PrEP and PEP, respectively. Most believed that pharmacist-initiated PrEP/PEP is important (96%) and were willing to provide PrEP (81%); fewer (27%) had PrEP/PEP training. Common implementation barriers were lack of staff/time and payment for pharmacist services. Participants preferred PrEP implementation models with in-pharmacy rapid oral HIV testing and pharmacists specifically hired to provide PrEP services.

**Conclusions:**

Despite pharmacists’ supportive attitudes, Senate Bill 159 implementation in California pharmacies remains limited, in part due to policy-level and organizational-level barriers. Ensuring PrEP/PEP-related payment for services and sufficient workforce capacity is key to leveraging pharmacists’ role in HIV prevention.

## Teaser

Despite enabling state legislation and high willingness to provide pre-exposure prophylaxis (PrEP), few (11%) participants in the California Pharmacist Study (2022) reported that pharmacists at their pharmacy currently initiate PrEP. Ensuring payment for services and enhanced workforce capacity could facilitate wider implementation.

In 2019, there were an estimated 34 800 new human immunodeficiency virus (HIV) infections in the United States (US) and more than 4500 new HIV diagnoses in California alone [1, 2]. The US HIV epidemic disproportionately affects men who have sex with men, for whom the estimated lifetime risk of HIV diagnosis is 1 in 6, and is defined by persistent racial/ethnic disparities that disproportionately affect Black and Latina/o people [3]. Pre-exposure prophylaxis (PrEP) and post-exposure prophylaxis (PEP) are highly effective HIV prevention tools, yet uptake remains low and may be hindered by intersectional stigma and other access barriers related to racism, homophobia, and structural inequity [4–10].

The US National HIV/AIDS Strategy highlights the opportunity to leverage pharmacists’ role as community healthcare providers to expand access to HIV prevention, including PrEP [11]. Almost 90% of the US population lives within 5 miles of a community pharmacy [12], and pharmacists are highly trained medical professionals who are well-suited to independently provide PrEP services when enabled through state legislation. Indeed, past studies have found high support for and interest in pharmacist-initiated PrEP among both pharmacists and potential PrEP users [13–15].

To reduce barriers to PrEP and PEP, California's Senate Bill (SB) 159 was passed in 2019, permitting pharmacists to independently prescribe PEP and up to 60 days of PrEP to clients with a recent negative HIV test [16] (in the context of this study, “prescribing” refers to pharmacists issuing prescriptions for medications, including PrEP and PEP, most often under authority granted by a statewide protocol; in California, this is also known as “furnishing”). Implementation became possible in late 2020 following protocol development and launch of the required training program [17]. Although timely evaluation of SB 159's implementation is critical to understand its impact, no studies have examined implementation within a broad range of pharmacy settings across the state [18]. California's experiences as the first state to allow pharmacist-initiated PrEP may also have broader relevance as a roadmap for other states in which similar legislation has recently been enacted [19]. Therefore, we surveyed California pharmacists about pharmacist-initiated PrEP and PEP provision 2 years after SB 159 implementation began.

## METHODS

From October through December 2022, we conducted a cross-sectional online survey of California pharmacists and pharmacy students, primarily recruited via email promotion by 2 professional organizations for California pharmacists. Eligible participants were (1) at least 18 years old, (2) a pharmacist or pharmacy student, (3) currently residing in California, and (4) willing to provide informed consent. Participants had the option to provide a name and email address to receive compensation in the form of a $20 gift card and/or entry into prize drawings. Participants who declined to provide contact information remained anonymous.

Participants completed a Qualtrics-based survey assessing the implementation of pharmacist-initiated PrEP/PEP, including knowledge, attitudes, and barriers. In exploratory analyses, we estimated prevalence ratios (PRs) comparing implementation by pharmacy characteristics via log-binomial regression in R, version 4.2.1 [20].

To elicit implementation preferences, we also administered a discrete choice experiment (DCE), a stated choice method with predictive value for health-related behavior [21]. Participants were presented with pairs of PrEP implementation scenarios that varied on 4 attributes (HIV testing procedures, how services fit into the pharmacy workflow, eligibility screening and counseling procedures, and maximum dispensing period before referral), each with 3–4 possible levels, and chose the scenario from each pair that they would prefer for implementation in their pharmacy. Participants were block-randomized to receive 4 of 16 possible pairs to choose between (eg, Supplementary Figure 1) [22].

To identify implementation characteristics shaping participants’ choices, we fit McFadden's conditional logit choice model in Stata software, version 17 [23]. The resulting effects-coded model “preference weights” represent the strength of participants’ preferences for scenarios with specific attribute levels estimated relative to the mean effect of the given attribute across levels [24]. A positive preference weight for a given attribute level suggests that scenarios with this level were more likely to be chosen; the reverse is true for a negative preference weight.

### Patient Consent Statement

This study was approved by the Office of the Human Research Protection Program at the University of California, Los Angeles with the University of California, Berkeley Committee for Protection of Human Subjects in reliance. We obtained a waiver of written consent; all participants provided informed consent in Qualtrics before completing the survey.

## RESULTS

Of 2633 survey responses, 919 (35%) were unique, valid participants. Participants most often reported currently or most recently working in community (43%), hospital (28%), or clinic or ambulatory care (16%) settings (Table 1). Among participants at community pharmacies, 59% worked in chains (national or state); 38% worked in independent pharmacies.

**Table 1.** Participant Characteristics, Knowledge of HIV Pre-exposure Prophylaxis and Post-exposure Prophylaxis, and Pharmacy Implementation of California's Senate Bill 159 Stratified by Pharmacy Setting in the California Pharmacist Study, 2022

Characteristic	Community (n = 393)	Hospital (n = 255)	Clinic or Ambulatory (n = 143)	Other (n = 128)	Overall (N = 919)
Age in years, mean ± SD	39.8 ± 13.7	37.1 ± 11.6	40.4 ± 12.9	39.1 ± 12.4	39.1 ± 12.9
Gender					
Cisgender man	138 (38.9)	74 (33.6)	43 (34.1)	34 (30.1)	289 (35.5)
Cisgender woman	214 (60.3)	145 (65.9)	83 (65.9)	76 (67.3)	518 (63.6)
Nonbinary or transgender of any gender identity	3 (0.8)	1 (0.5)	0 (0.0)	3 (2.7)	7 (0.9)
Race and ethnicity					
American Indian or Alaska Native	0 (0.0)	2 (0.9)	0 (0.0)	2 (1.9)	4 (0.5)
Asian	209 (61.3)	153 (72.2)	68 (60.2)	67 (62.6)	497 (64.3)
Black or African American	7 (2.1)	4 (1.9)	4 (3.5)	0 (0.0)	15 (1.9)
Hispanic or Latino	14 (4.1)	6 (2.8)	8 (7.1)	8 (7.5)	36 (4.7)
Native Hawaiian or Pacific Islander	1 (0.3)	0 (0.0)	0 (0.0)	0 (0.0)	1 (0.1)
White	96 (28.2)	37 (17.5)	28 (24.8)	23 (21.5)	184 (23.8)
Multiracial	8 (2.3)	4 (1.9)	2 (1.8)	3 (2.8)	17 (2.2)
Other	6 (1.8)	6 (2.8)	3 (2.7)	4 (3.7)	19 (2.5)
Pharmacist category					
Currently practicing licensed pharmacist	316 (80.4)	221 (86.7)	127 (88.8)	105 (82.0)	769 (83.7)
Pharmacy student	49 (12.5)	19 (7.5)	6 (4.2)	9 (7.0)	83 (9.0)
Retired pharmacist	16 (4.1)	11 (4.3)	3 (2.1)	4 (3.1)	34 (3.7)
Other nonpracticing pharmacist	12 (3.1)	4 (1.6)	7 (4.9)	10 (7.8)	33 (3.6)
Heard of HIV PrEP					
Yes	354 (90.1)	241 (94.5)	132 (92.3)	114 (89.1)	841 (91.5)
No	33 (8.4)	10 (3.9)	9 (6.3)	13 (10.2)	65 (7.1)
Not sure/don't know	6 (1.5)	4 (1.6)	2 (1.4)	1 (0.8)	13 (1.4)
Heard of HIV PEP					
Yes	354 (90.1)	245 (96.1)	134 (93.7)	117 (91.4)	850 (92.5)
No	29 (7.4)	6 (2.4)	7 (4.9)	10 (7.8)	52 (5.7)
Not sure/don't know	10 (2.5)	4 (1.6)	2 (1.4)	1 (0.8)	17 (1.8)
Heard of Senate Bill 159					
Yes	288 (73.3)	177 (69.4)	109 (76.2)	88 (68.8)	662 (72.0)
No	105 (26.7)	78 (30.6)	34 (23.8)	40 (31.2)	257 (28.0)
Received training on providing PrEP/PEP					
Yes, completed training	100 (25.4)	49 (19.2)	29 (20.3)	21 (16.4)	199 (21.7)
Yes, training in progress	23 (5.9)	14 (5.5)	7 (4.9)	5 (3.9)	49 (5.3)
No	270 (68.7)	192 (75.3)	107 (74.8)	102 (79.7)	671 (73.0)
Pharmacists at pharmacy currently initiate HIV PrEP as authorized by SB 159					
Yes	44 (11.2)	26 (10.2)	17 (11.9)	9 (7.0)	96 (10.4)
No	285 (72.5)	172 (67.5)	80 (55.9)	86 (67.2)	623 (67.8)
Not sure/don't know	64 (16.3)	57 (22.4)	33 (23.1)	14 (10.9)	168 (18.3)
NA (never worked in a pharmacy)	0 (0.0)	0 (0.0)	13 (9.1)	19 (14.8)	32 (3.5)
Pharmacists at pharmacy currently provide HIV PEP as authorized by SB 159					
Yes	46 (11.7)	41 (16.1)	17 (11.9)	12 (9.4)	116 (12.6)
No	280 (71.4)	151 (59.2)	75 (52.4)	85 (66.4)	591 (64.4)
Not sure/don't know	66 (16.8)	63 (24.7)	39 (27.3)	13 (10.2)	181 (19.7)
NA (never worked in a pharmacy)	0 (0.0)	0 (0.0)	12 (8.4)	18 (14.1)	30 (3.3)

Data are presented as No. (column %) unless otherwise stated, excluding missing responses (n = 76 age, n = 105 gender, n = 146 race/ethnicity, n = 1 currently provide HIV PEP).

Abbreviations: HIV, human immunodeficiency virus; NA, not applicable; PEP, post-exposure prophylaxis; PrEP, pre-exposure prophylaxis; SB 159, Senate Bill 159; SD, standard deviation.

Most participants had heard of PrEP and PEP (92% each) and SB 159 (72%), while 27% had training on providing PrEP/PEP in a pharmacy setting. Participants who worked in community pharmacies were more likely to report having training compared to those in other settings (31% vs 24%; PR, 1.3 [95% confidence interval {CI}, 1.1–1.6]).

Among respondents who had ever worked in any pharmacy setting (97%), 11% and 13% reported that pharmacists at their pharmacy initiate PrEP and PEP as authorized by SB 159, respectively. Almost 20% were unsure. When comparing implementation by pharmacy characteristics (excluding unsure responses), participants at chain community pharmacies more often reported PrEP provision than those at independent community pharmacies (17% vs 9%; PR, 2.0 [95% CI, 1.1–3.7]), while participants in hospital settings more often reported PEP provision than those in community settings (21% vs 14%; PR, 1.5 [95% CI, 1.0–2.2]) (Supplementary Table 1). Participants from pharmacies located outside of Southern California were less likely to report PrEP provision (eg, 9% in the San Francisco Bay Area vs 19% in Los Angeles County; PR, 0.5 [95% CI, 0.3–0.9]).

**Figure 1.**
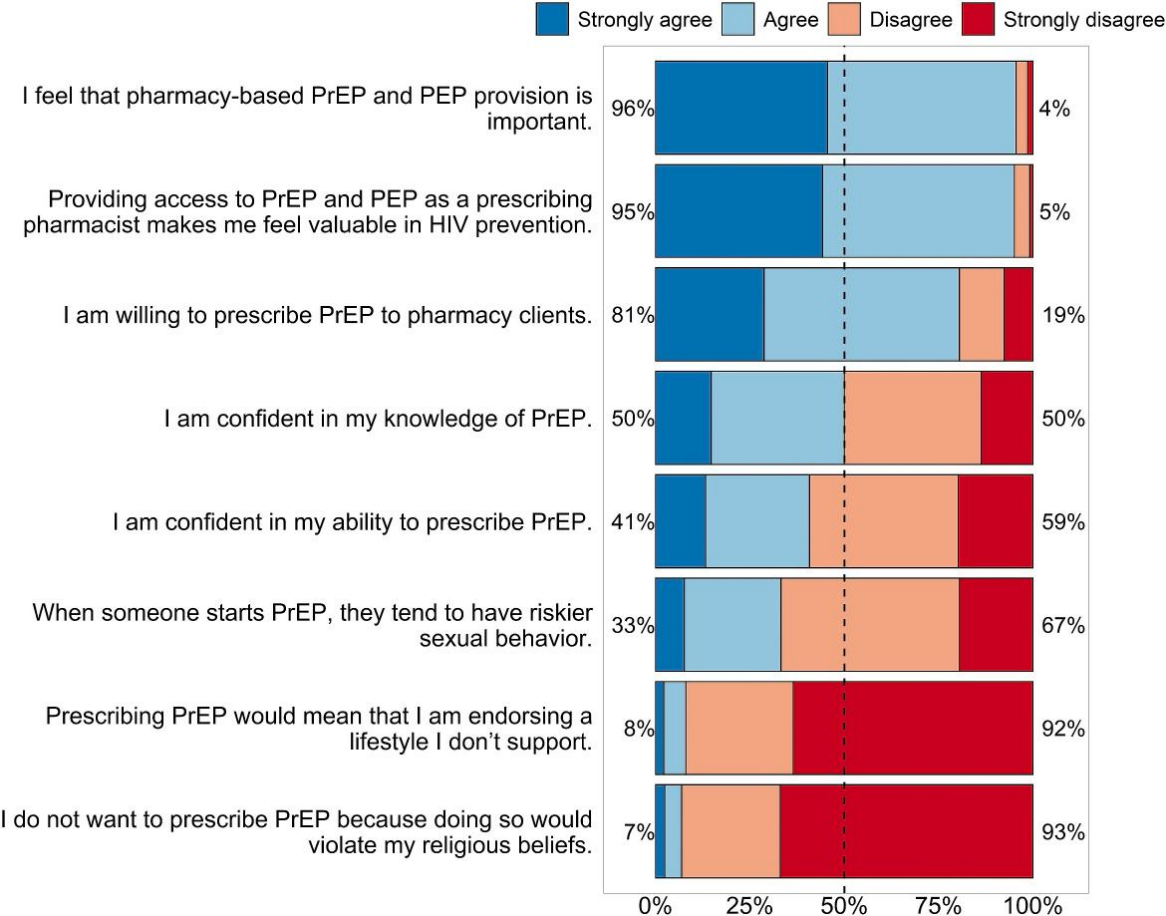
Attitudes about pre-exposure prophylaxis (PrEP) and post-exposure prophylaxis (PEP) in the California Pharmacist Study, 2022. Percentages are the sum of “strongly agree” and “agree” (left-hand side) or the sum of “strongly disagree” and “disagree” (right-hand side), excluding missing and “not applicable” responses (see Supplementary Table 2).

Most participants agreed that pharmacy-based PrEP and PEP provision is important (96%) and expressed willingness to prescribe PrEP to pharmacy clients (81%) (Figure 1, Supplementary Table 2). Fewer participants were confident in their knowledge of PrEP (50%) or their ability to prescribe PrEP (41%). Less than 10% expressed moral or religious objections to providing PrEP.

**Figure 2.**
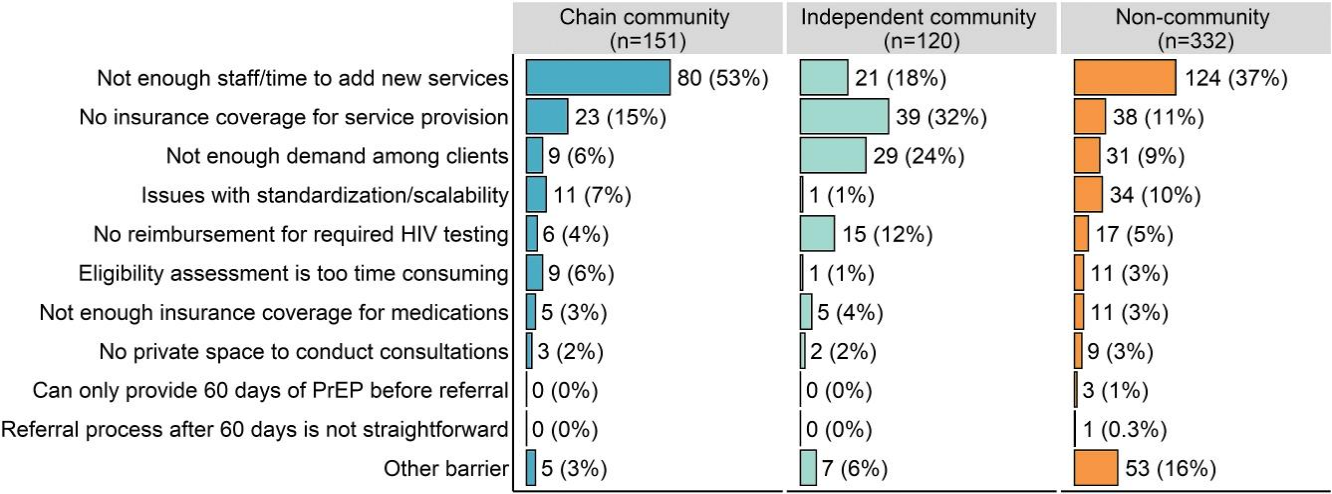
Main barriers to implementing pharmacist-initiated pre-exposure prophylaxis (PrEP) provision in the California Pharmacist Study, 2022. Data are No. (column %) among n = 603 participants whose pharmacy does not provide PrEP under Senate Bill 159, excluding missing responses (n = 12) and participants from community pharmacies of unspecified type (n = 8).

Participants at pharmacies that did not offer pharmacist-prescribed PrEP most often identified insufficient staff/time to add new services or lack of insurance coverage for service provision as the main barrier to implementation (Figure 2, Supplementary Table 3). Insufficient staff/time was more often selected as the main barrier to PrEP implementation by participants at chain community pharmacies than by those at independent community pharmacies (53% vs 18%; PR, 3.0 [95% CI, 2.0–4.6]). Participants at independent community pharmacies most frequently cited lack of insurance coverage (32%) and low client demand (24%). Findings were similar for PEP implementation (Supplementary Figure 2, Supplementary Table 3).

**Figure 3.**
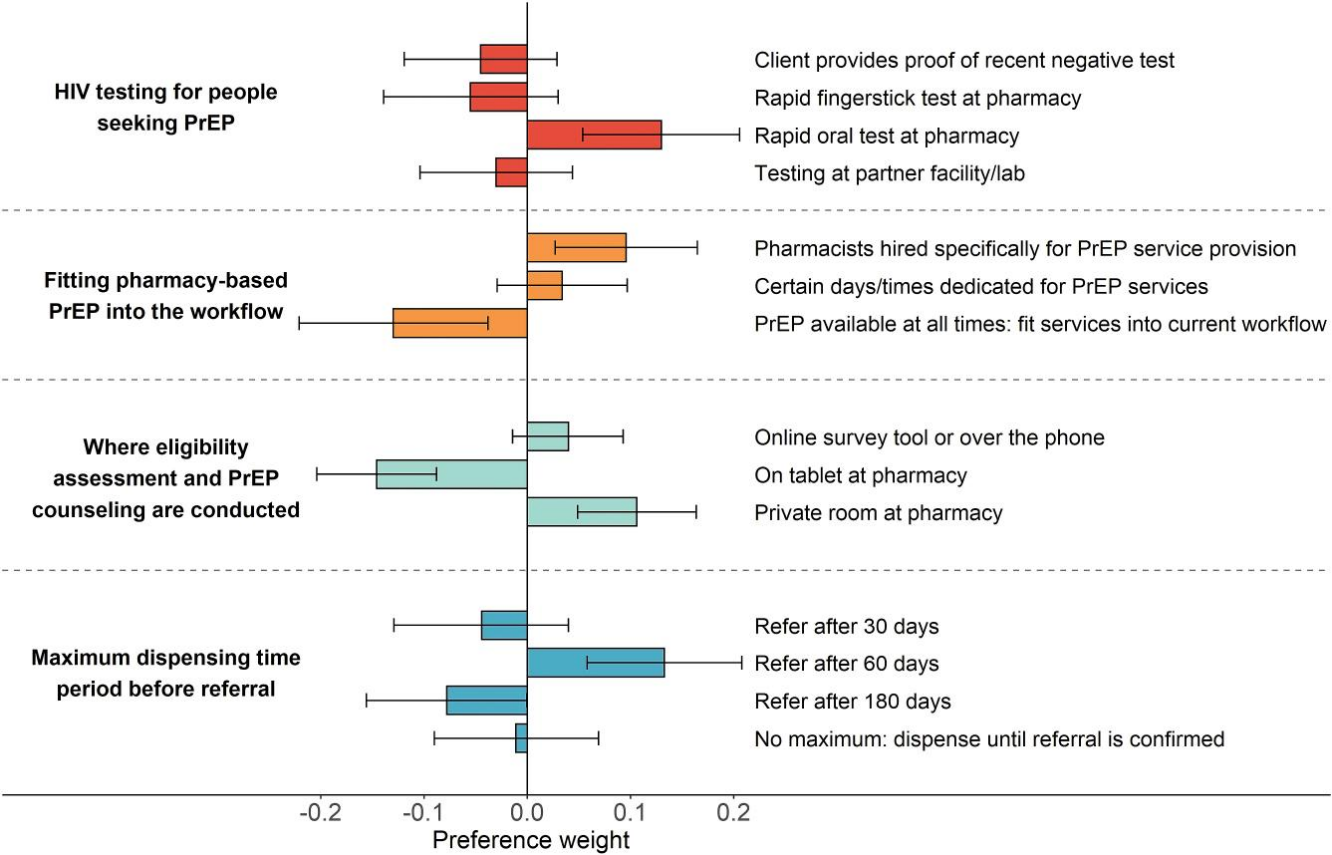
Pre-exposure prophylaxis implementation preferences assessed via discrete choice experiment in the California Pharmacist Study, 2022. Effects-coded preference weights and 95% confidence intervals estimated via McFadden's conditional logit choice model among 876 participants who completed ≥1 choice task question. Abbreviations: HIV, human immunodeficiency virus: PrEP, pre-exposure prophylaxis.

In the DCE, 95% of participants (n = 876) responded to at least 1 of 4 questions in which they chose between pairs of pharmacy-based PrEP implementation scenarios, completing 3481 DCE questions in total (mean number of questions completed per participant: 3.8). Analyses revealed that participants preferred implementation scenarios that included in-pharmacy rapid oral HIV testing (vs in-pharmacy rapid fingerstick testing or testing in another setting), pharmacists hired specifically for PrEP service provision (vs fitting services into the current workflow), counseling conducted in a private room (vs conducted on a tablet), and/or referral after 60 days of PrEP dispensing (vs referral after 30 days or 180 days) (Figure 3, Supplementary Table 4).

## DISCUSSION

Our survey found high willingness to prescribe PrEP among California pharmacists, yet low implementation of pharmacist-initiated PrEP and PEP 2 years after the practice was enabled. Although pharmacists overwhelmingly considered pharmacy-based HIV prevention to be important, they reported barriers to implementation including staff/time constraints, lack of insurance coverage for service provision, and low perceived demand among clients. These barriers varied by pharmacy setting, with staff/time constraints more often reported by respondents at chain than independent community pharmacies.

Implementation preferences revealed via DCE suggest that pharmacists may prefer to be actively engaged in the PrEP provision process (eg, in-pharmacy rapid oral HIV testing over testing at another facility). Participants’ preference for hiring pharmacists specifically for PrEP services, rather than incorporating services into their current workflow, aligns with staff/time barriers identified elsewhere in the survey, underscoring the importance of increasing workforce capacity to accommodate new service provision. Finally, participants’ preference for the current 60-day PrEP referral period, rather than an expanded 180-day period, may relate to perceived challenges in making successful referrals as the period lengthens or may in part reflect familiarity with the 60-day period encoded in law. Notably, when asked earlier in the survey, many pharmacists disagreed that the 60-day period was sufficient to ensure referral.

To our knowledge, this study is the first to evaluate SB 159 implementation statewide. One prior study found that only 3% of San Francisco Bay Area community and mail order pharmacies offered pharmacist-prescribed PrEP or PEP in April 2021, less than a year after enactment [18]. The present study updates and expands upon these findings by surveying pharmacists from a broad range of pharmacy settings (eg, hospital and outpatient clinics) across all regions of the state. Our findings are similar to earlier studies reporting a lag in pharmacy implementation for expanded scope of practice across multiple service areas [25, 26]. The implementation barriers observed overlap with those reported in qualitative studies of California pharmacists (eg, payment for services, limited staff time, and low client awareness) [18, 27]. Another recent survey also found that community pharmacists at chain pharmacies are more likely than those at independent pharmacies to report that staff/time constraints impede clinical service provision [28], indicating that structural and organizational differences between these settings may influence the adoption of initiatives to expand access to health services.

This study provides a comprehensive assessment of pharmacist-initiated PrEP/PEP provision at a critical juncture of SB 159 implementation. As is true for all surveys based on self-report, there is potential for response bias. We recruited participants primarily via professional organizations and used rigorous best practice procedures to ensure data integrity, likely bolstering the validity of responses. We aimed to assess the experiences of participating pharmacists, rather than the proportion of *pharmacies* offering PrEP/PEP services. Still, participants’ pharmacies were located in >450 unique California ZIP codes, reducing the likelihood of substantial overlap in their pharmacies. The demographics of our convenience sample are similar to external data about California pharmacists and pharmacy students (Supplementary Table 5) and largely reflect the state's population distribution geographically (Supplementary Figure 3), but it remains possible that enrollment rates varied based on other characteristics associated with implementation outcomes. Additionally, the relatively small number of rural participants and participants of certain racial or ethnic identities (eg, Black, Indigenous, Latina/o) precludes subgroup analyses and represents an important avenue for future research. While this study provides necessary insight into pharmacists’ perspectives around PrEP/PEP provision, a holistic understanding of both provider- *and* client-side experiences is essential to inform accessible PrEP/PEP implementation models in pharmacies.

In conclusion, despite California's legislative efforts to enable pharmacist-initiated PrEP/PEP and supportive attitudes among pharmacists, current levels of implementation fall short of the vision for significantly expanded access. Further efforts to ensure payment for service provision and sufficient workforce capacity are needed to make broader implementation feasible. This study may have national relevance in light of the 2022–2025 US National HIV/AIDS Strategy's inclusion of pharmacists as key providers of HIV prevention and multiple states’ recent expansion of pharmacists’ scope of practice to include PrEP provision [11, 19]. Our findings are particularly timely and relevant to current policymaking, including the US Centers for Medicare and Medicaid Services’ recent proposal to cover PrEP medications and related counseling services as preventive care and pending California legislation that would require health plans to pay for pharmacists’ services during PrEP/PEP provision [29, 30].

## Supplementary Material

ofad549_Supplementary_Data

## Supplementary Data

Supplementary materials are available at *Open Forum Infectious Diseases* online. Consisting of data provided by the authors to benefit the reader, the posted materials are not copyedited and are the sole responsibility of the authors, so questions or comments should be addressed to the corresponding author.

## Notes

***Acknowledgments.*** We are grateful to Donald Kishi, Craig Pulsipher, Dorie Apollonio, Jerika Lam, Kim Koester, Tam Phan, Robert Gamboa, Richard Salazar, and Amanda Mazur for their contributions. The study was made possible through the recruitment support of the California Society of Health-System Pharmacists and the California Pharmacists Association.

***Data availability.*** Data are available upon reasonable request.

***Financial support*.** The California Pharmacist Study was supported by the California HIV/AIDS Research Program; the University of California Office of the President (H21PC3238 to S.I.M. and H21PC3466 to A.M.O.); and the UCLA Center on Reproductive Health, Law, and Policy.
