# Supplementary material for: Opportunities to Increase Access to HIV Prevention: Evaluating the Implementation of Pharmacist-Initiated Pre-exposure Prophylaxis in California
Source: Open Forum Infect Dis. 2023 Nov 3;10(11):ofad549. doi: 10.1093/ofid/ofad549 (PMC10651201; doi:10.1093/ofid/ofad549)
Supplement: ofad549_Supplementary_Data [file ofad549_supplementary_data.docx]

**Supplemental Material**

[Figure S1. Example discrete choice experiment question in the California Pharmacist Study. 2](#_Toc144207022)

[Table S1. Implementation of Senate Bill (SB) 159 by pharmacy setting in the California Pharmacist Study, 2022. 3](#_Toc144207023)

[Table S2. Attitudes about PrEP and PEP in the California Pharmacist Study, 2022. 4](#_Toc144207024)

[Table S3. Main barriers to PrEP and PEP provision under SB 159 by pharmacy setting in the California Pharmacist Study, 2022. 6](#_Toc144207025)

[Figure S2. Main barriers to PEP provision under SB 159 by pharmacy setting in the California Pharmacist Study, 2022. 7](#_Toc144207026)

[Table S4. Preference weights for PrEP implementation attributes estimated via discrete choice experiment in the California Pharmacist Study, 2022. 8](#_Toc144207027)

[Table S5. Demographics of participants in the California Pharmacist Study (2022) compared to external data (2013–2019). 9](#_Toc144207028)

[Figure S3. Locations of participants’ current or most recent pharmacy workplace in the California Pharmacist Study (2022) compared to county populations (2015 U.S. census). 10](#_Toc144207029)

# Figure S1. Example discrete choice experiment question in the California Pharmacist Study.

**
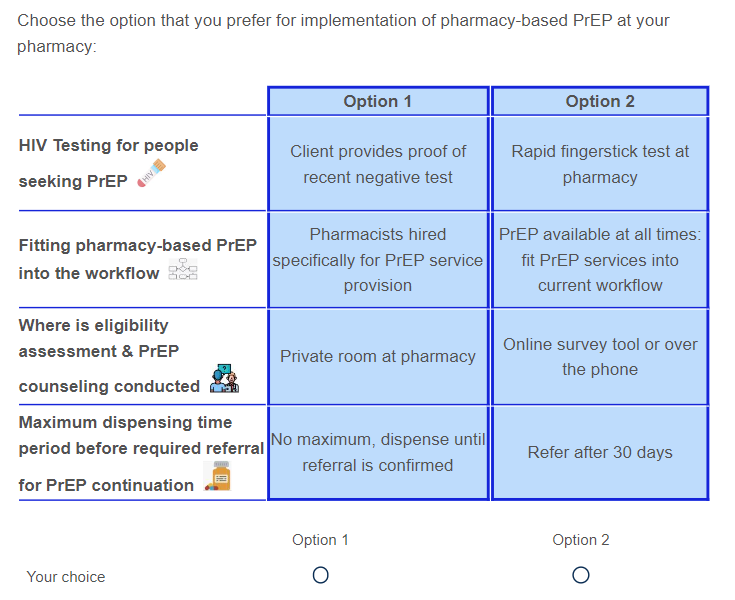
**

# Table S1. Implementation of Senate Bill (SB) 159 by pharmacy setting in the California Pharmacist Study, 2022.

|  | **Pharmacists at pharmacy currently initiate HIV PrEP as authorized by SB 159*** | | | **Pharmacists at pharmacy currently provide HIV PEP as authorized by SB 159*** | | |
| --- | --- | --- | --- | --- | --- | --- |
|  | Yes,  n (row %) | No,  n (row %) | PR (95% CI) | Yes,  n (row %) | No,  n (row %) | PR (95% CI) |
| **Overall** | 96 (13.4) | 623 (86.6) | - | 116 (16.4) | 591 (83.6) | - |
| **Pharmacy setting** |  |  |  |  |  |  |
| Community | 44 (13.4) | 285 (86.6) | Reference | 46 (14.1) | 280 (85.9) | Reference |
| Hospital | 26 (13.1) | 172 (86.9) | 0.98 (0.63, 1.54) | 41 (21.4) | 151 (78.6) | 1.51 (1.03, 2.22) |
| Clinic or ambulatory care | 17 (17.5) | 80 (82.5) | 1.31 (0.79, 2.19) | 17 (18.5) | 75 (81.5) | 1.31 (0.79, 2.17) |
| Other | 9 (9.5) | 86 (90.5) | 0.71 (0.36, 1.40) | 12 (12.4) | 85 (87.6) | 0.88 (0.48, 1.59) |
| **Community pharmacy type** |  |  |  |  |  |  |
| Chain | 32 (17.4) | 152 (82.6) | 1.97 (1.05, 3.68) | 32 (17.5) | 151 (82.5) | 1.67 (0.93, 3.01) |
| Independent | 12 (8.8) | 124 (91.2) | Reference | 14 (10.4) | 120 (89.6) | Reference |
| **Pharmacy census region** |  |  |  |  |  |  |
| Los Angeles County | 39 (18.7) | 170 (81.3) | Reference | 42 (20.8) | 160 (79.2) | Reference |
| San Francisco Bay Area | 14 (9.3) | 136 (90.7) | 0.50 (0.28, 0.89) | 22 (15.1) | 124 (84.9) | 0.72 (0.45, 1.16) |
| San Diego - Imperial | 10 (13.3) | 65 (86.7) | 0.71 (0.38, 1.36) | 8 (10.8) | 66 (89.2) | 0.52 (0.26, 1.06) |
| Orange County | 13 (20.3) | 51 (79.7) | 1.09 (0.62, 1.91) | 13 (20.3) | 51 (79.7) | 0.98 (0.56, 1.70) |
| Superior California | 6 (10.2) | 53 (89.8) | 0.54 (0.24, 1.22) | 7 (12.1) | 51 (87.9) | 0.58 (0.28, 1.22) |
| Other regions**^†^** | 12 (8.3) | 132 (91.7) | 0.45 (0.24, 0.82) | 22 (15.2) | 123 (84.8) | 0.73 (0.46, 1.17) |

PR: prevalence ratio estimated via log-binomial regression, CI: confidence interval.

*Excludes participants who responded “don’t know” (PrEP n=168, PEP n=181) or “N/A - never worked in a pharmacy” (PrEP n=32, PEP n=30). Bivariate comparisons also exclude missing independent variable responses (n=9 community pharmacy type, n=18 pharmacy census region).

**^†^**Other regions: Inland Empire n=43, Southern San Joaquin Valley n=29, Central Coast n=28, Northern San Joaquin Valley n=23, North Coast n=21.

# Table S2. Attitudes about PrEP and PEP in the California Pharmacist Study, 2022.

|  | **N=919** |
| --- | --- |
| **I am confident in my knowledge of PrEP.** |  |
| Strongly agree | 126 (13.7) |
| Agree | 302 (32.9) |
| Disagree | 311 (33.8) |
| Strongly disagree | 117 (12.7) |
| N/A | 63 (6.9) |
| **I am confident in my ability to prescribe PrEP.** |  |
| Strongly agree | 110 (12.0) |
| Agree | 228 (24.8) |
| Disagree | 326 (35.5) |
| Strongly disagree | 164 (17.8) |
| N/A | 91 (9.9) |
| **I am willing to prescribe PrEP to pharmacy clients.** |  |
| Strongly agree | 227 (24.7) |
| Agree | 409 (44.5) |
| Disagree | 94 (10.2) |
| Strongly disagree | 60 (6.5) |
| N/A | 129 (14.0) |
| **I feel that pharmacy-based PrEP and PEP provision is important.** | |
| Strongly agree | 397 (43.2) |
| Agree | 435 (47.3) |
| Disagree | 27 (2.9) |
| Strongly disagree | 12 (1.3) |
| N/A | 48 (5.2) |
| **When someone starts PrEP, they tend to have riskier sexual behavior.** | |
| Strongly agree | 61 (6.6) |
| Agree | 204 (22.2) |
| Disagree | 377 (41.0) |
| Strongly disagree | 155 (16.9) |
| N/A | 122 (13.3) |
| **Providing access to PrEP and PEP as a prescribing pharmacist makes me feel valuable in HIV prevention.** | |
| Strongly agree | 370 (40.3) |
| Agree | 426 (46.4) |
| Disagree | 34 (3.7) |
| Strongly disagree | 7 (0.8) |
| N/A | 81 (8.8) |
| **I do not want to prescribe PrEP because doing so would violate my religious beliefs.** | |
| Strongly agree | 20 (2.2) |
| Agree | 36 (3.9) |
| Disagree | 212 (23.1) |
| Strongly disagree | 543 (59.1) |
| N/A | 108 (11.8) |
| **Prescribing PrEP would mean that I am endorsing a lifestyle I don’t support.** | |
| Strongly agree | 18 (2.0) |
| Agree | 49 (5.3) |
| Disagree | 238 (25.9) |
| Strongly disagree | 532 (57.9) |
| N/A | 82 (8.9) |

n (column %), excluding missing responses (n=1 valuable in HIV prevention).

# Table S3. Main barriers to PrEP and PEP provision under SB 159 by pharmacy setting in the California Pharmacist Study, 2022.

|  | **Chain community** | **Independent community** | **Other setting*** | **Overall^†^** |
| --- | --- | --- | --- | --- |
| **Main barrier to implementing pharmacist-initiated PrEP** | **n=151** | **n=120** | **n=340** | **n=611** |
| Not enough staff/time to add new services | 80 (53.0) | 21 (17.5) | 128 (37.6) | 229 (37.5) |
| No insurance coverage provided for furnishing PrEP (medication is  covered but service is not) | 23 (15.2) | 39 (32.5) | 41 (12.1) | 103 (16.9) |
| Not enough demand for PrEP among clients | 9 (6.0) | 29 (24.2) | 32 (9.4) | 70 (11.5) |
| Issues with standardization and scalability | 11 (7.3) | 1 (0.8) | 34 (10.0) | 46 (7.5) |
| No reimbursement provided for the required HIV testing | 6 (4.0) | 15 (12.5) | 17 (5.0) | 38 (6.2) |
| Eligibility assessment for PrEP is too time consuming | 9 (6.0) | 1 (0.8) | 11 (3.2) | 21 (3.4) |
| Not enough insurance coverage for PrEP medications | 5 (3.3) | 5 (4.2) | 11 (3.2) | 21 (3.4) |
| No private space to conduct consultations | 3 (2.0) | 2 (1.7) | 9 (2.6) | 14 (2.3) |
| Can only provide 60 days of PrEP before a referral is required | 0 (0.0) | 0 (0.0) | 3 (0.9) | 3 (0.5) |
| The provider referral process after the 60-day window is not  straightforward | 0 (0.0) | 0 (0.0) | 1 (0.3) | 1 (0.2) |
| Other barrier | 5 (3.3) | 7 (5.8) | 53 (15.6) | 65 (10.6) |
| **Main barrier to implementing pharmacist-prescribed PEP** | **n=150** | **n=116** | **n=313** | **n=579** |
| Not enough staff/time to add new services | 82 (54.7) | 20 (17.2) | 116 (37.1) | 218 (37.7) |
| No insurance coverage provided for furnishing PEP (medication is  covered but service is not) | 16 (10.7) | 39 (33.6) | 35 (11.2) | 90 (15.5) |
| Not enough demand for PEP among clients | 14 (9.3) | 26 (22.4) | 41 (13.1) | 81 (14.0) |
| Issues with standardization and scalability | 8 (5.3) | 7 (6.0) | 27 (8.6) | 42 (7.3) |
| No reimbursement provided for the required HIV testing | 9 (6.0) | 12 (10.3) | 8 (2.6) | 29 (5.0) |
| Not enough insurance coverage for PEP medications | 2 (1.3) | 3 (2.6) | 10 (3.2) | 15 (2.6) |
| Monitoring patients on the PEP regimen is too complicated | 5 (3.3) | 1 (0.9) | 8 (2.6) | 14 (2.4) |
| No private space to conduct consultations | 5 (3.3) | 1 (0.9) | 8 (2.6) | 14 (2.4) |
| Eligibility assessment for PEP is too time consuming | 3 (2.0) | 3 (2.6) | 7 (2.2) | 13 (2.2) |
| Other barrier | 6 (4.0) | 4 (3.4) | 53 (16.9) | 63 (10.9) |

n (column %) unless otherwise stated, excluding missing responses (n=12).

*Includes n=8 participants from community pharmacies of unspecified type.

**^†^**Question(s) asked of participants who reported that their pharmacy does not initiate PrEP and/or provide PEP as authorized by Senate Bill 159.

# Figure S2. Main barriers to PEP provision under SB 159 by pharmacy setting in the California Pharmacist Study, 2022.


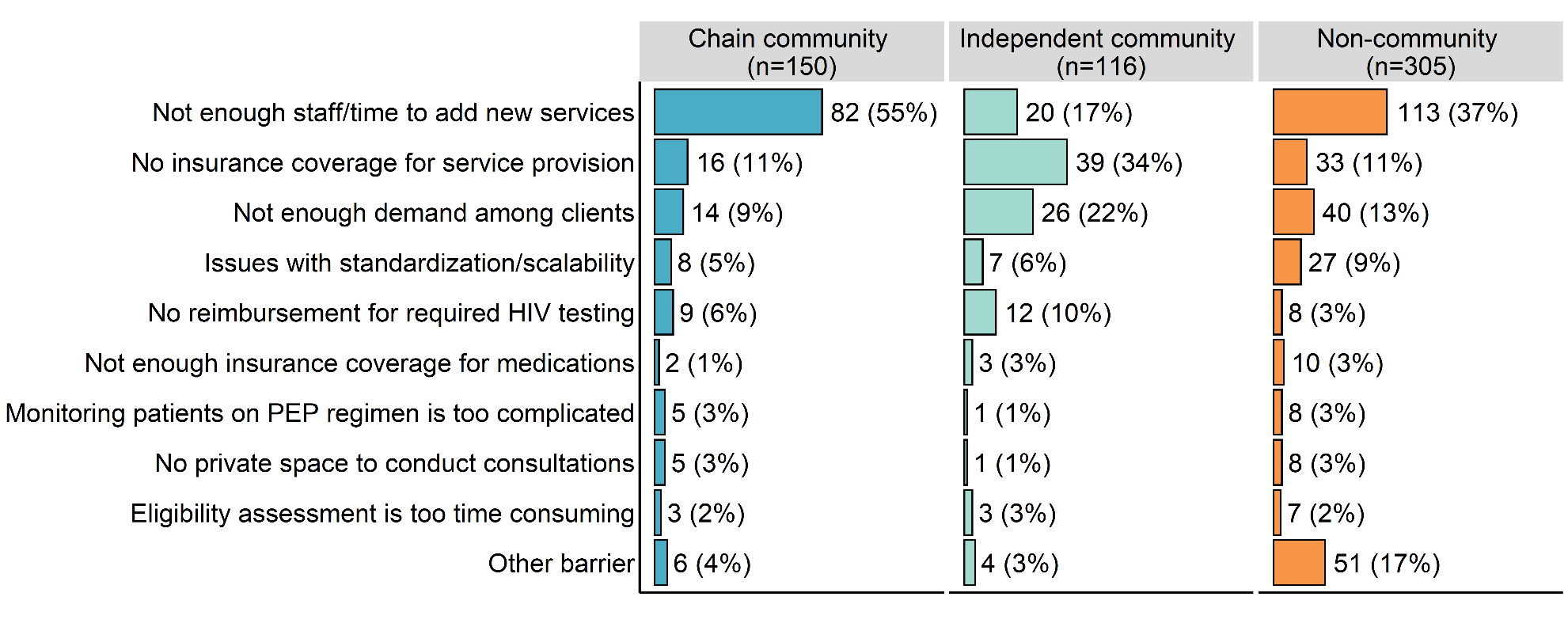


n (column %) among n=571 participants whose pharmacy does not provide PEP under SB 159, excluding missing responses (n=12) and participants from community pharmacies of unspecified type (n=8).

# Table S4. Preference weights for PrEP implementation attributes estimated via discrete choice experiment in the California Pharmacist Study, 2022.

|  | **Preference weight (95% CI)** |
| --- | --- |
| **HIV testing for people seeking PrEP** |  |
| Client provides proof of recent negative test | -0.045 (-0.119, 0.029) |
| Rapid fingerstick test at pharmacy | -0.055 (-0.139, 0.030) |
| Rapid oral test at pharmacy | 0.130 (0.054, 0.206) |
| Testing at partner facility/lab | -0.030 (-0.104, 0.044) |
| **Fitting pharmacy-based PrEP into the workflow** |  |
| Pharmacists hired specifically for PrEP service provision | 0.096 (0.027, 0.165) |
| Certain days/times dedicated for PrEP services | 0.034 (-0.029, 0.097) |
| PrEP available at all times: fit PrEP services into current  workflow | -0.130 (-0.221, -0.038) |
| **Where eligibility assessment and PrEP counseling are conducted** | |
| Online survey tool or over the phone | 0.040 (-0.014, 0.093) |
| On tablet at pharmacy | -0.146 (-0.204, -0.088) |
| Private room at pharmacy | 0.106 (0.049, 0.164) |
| **Maximum dispensing time period before required referral for PrEP continuation** | |
| Refer after 30 days | -0.044 (-0.129, 0.040) |
| Refer after 60 days | 0.133 (0.058, 0.208) |
| Refer after 180 days | -0.078 (-0.156, -0.0003) |
| No maximum: dispense until referral is confirmed | -0.011 (-0.090, 0.069) |

CI: confidence interval.

Effects-coded preference weights and 95% confidence intervals estimated via McFadden’s conditional logit choice model among n=876 participants who completed ≥1 choice task question.

#

# Table S5. Demographics of participants in the California Pharmacist Study (2022) compared to external data (2013–2019).

|  | **2022 California Pharmacist Study (N=919)*** | **2013**–**2017 California pharmacists**** | **2019 California PharmD graduates***** |
| --- | --- | --- | --- |
| **Age in years (continuous), mean** | 39.1 | 42.9 | - |
| **Age in years (categorical), %** |  |  |  |
| Under 35 | 43.9 | 33.7 | - |
| 35–44 | 30.5 | 26.1 | - |
| 45–64 | 18.3 | 33.2 | - |
| 65 or older | 7.4 | 7.0 | - |
| **Women, %** | 63.6 | 61.6 | - |
| **Race and ethnicity, %** |  |  |  |
| American Indian or Alaska Native | 0.4 (0.5) | 0.03 | <1 |
| Asian | 54.1 (64.3) | 53.5 | 63 |
| Black or African American | 1.6 (1.9) | 2.5 | 3 |
| Hispanic or Latino | 3.9 (4.7) | 5.1 | 5 |
| Native Hawaiian or Pacific Islander | 0.1 (0.1) | 0.3 | <1 |
| White | 20.0 (23.8) | 35.5 | 19 |
| Multiracial | 1.8 (2.2) | 2.7 | 3 |
| Other | 2.1 (2.5) | 0.3 | - |
| Unknown or missing | 15.9 (-) | - | 5 |

*Percentages for age and gender exclude missing and ‘prefer not to specify’ responses (n=76 age, n=105 gender); percentages for race and ethnicity calculated both including and excluding missing and ‘prefer not to specify’ responses (n=146).

**U.S. Census Bureau, 2013-2017 American Communities Survey estimates as reported by the [Healthforce Center at UCSF](https://healthforce.ucsf.edu/sites/healthforce.ucsf.edu/files/publication-pdf/Pharmacist%20Workforce%20and%20Education%20in%20California.pdf) (2020). Percentage shown for Asian race and ethnicity is the sum of Asian (48.4%) and Filipino (5.1%) categories.

***U.S. Department of Education, 2019 IPEDS Completion Survey estimates as reported by the [California Health Care Foundation](https://www.chcf.org/wp-content/uploads/2021/03/HealthCareWorkforceAlmanac2021PharmacyQRG.pdf) (2021).

# Figure S3. Locations of participants’ current or most recent pharmacy workplace in the California Pharmacist Study (2022) compared to county populations (2015 U.S. census).


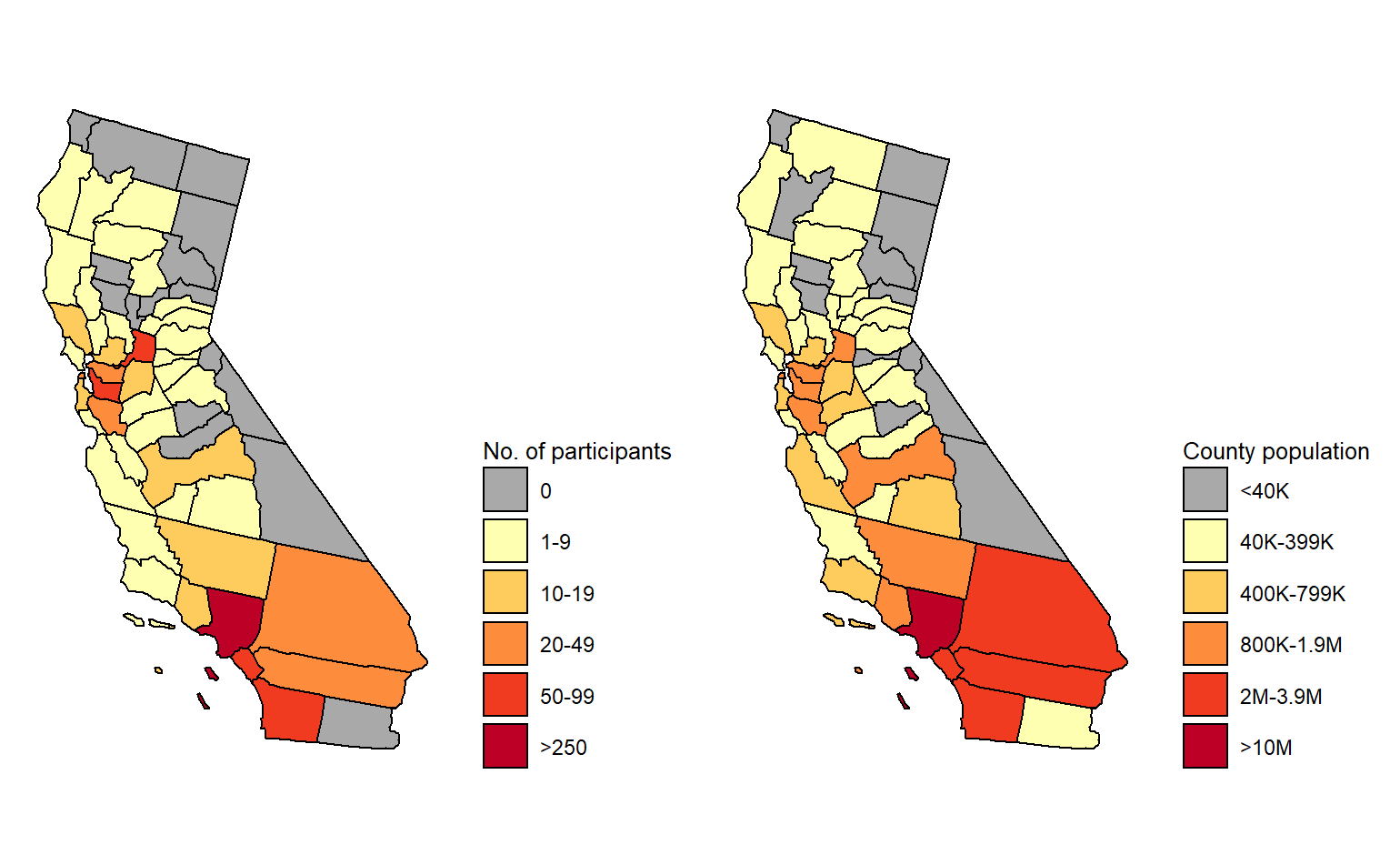


No.: number, K: thousand, M: million.

Left panel includes n=873 participants who provided a valid California ZIP code for their current or most recent pharmacy workplace (95% of study participants).

R packages: [usmap](https://cran.r-project.org/web/packages/usmap/usmap.pdf), [zipcodeR](https://cran.r-project.org/web/packages/zipcodeR/zipcodeR.pdf).
